# Enhancing Cross-Linking Efficiency in Gelatin-Based Hydrogels via Incorporation of Tannic Acid, Pluronic F-127, and Phytic Acid

**DOI:** 10.3390/polym17101372

**Published:** 2025-05-16

**Authors:** Njomza Ajvazi, Ingrid Milošev, Nataša Čelan Korošin, Peter Rodič, Bojan Božić

**Affiliations:** 1 Jožef Stefan Institute, Jamova 39, 1000 Ljubljana, Slovenia; ingrid.milosev@ijs.si (I.M.); peter.rodic@ijs.si (P.R.); 2 Valdoltra Orthopaedic Hospital, Jadranska c. 31, 6280 Ankaran, Slovenia; 3 Faculty of Chemistry and Chemical Technology, University of Ljubljana, Večna pot 113, 1000 Ljubljana, Slovenia; natasa.celan@fkkt.uni-lj.si; 4 Faculty of Biology, Institute of Physiology and Biochemistry “Ivan Djaja”, University of Belgrade, Studentski Trg 3, 11000 Belgrade, Serbia; bbozic@bio.bg.ac.rs

**Keywords:** hydrogels, gelatin, tannic acid, Pluronic F-127, phytic acid, biopolymers

## Abstract

This study enhanced the gelatin-based hydrogel formulation by incorporating tannic acid, triblock copolymer Pluronic F-127, and phytic acid to improve its physicochemical properties. The swelling behaviour of these hydrogels was evaluated in phosphate buffer solution at selected pH levels. Morphological and thermal properties of the investigated hydrogels were analysed using scanning electron microscopy, thermogravimetric analysis coupled with mass spectrometry, and differential scanning calorimetry. Fourier transform infrared spectroscopy was employed to investigate the chemical structure of the optimal hydrogel. Tannic acid was recognised as a key component responsible for significant improvements in the hydrogel’s overall properties, including greater swelling capacity in phosphate buffer solution, a more defined porous structure, enhanced thermal stability (with a melting point above physiological conditions), significantly increased mechanical strength, elasticity, and overall robustness, as well as stability of the hydrogel network structure. These enhancements make the gelatin-based hydrogels more suitable for biomedical applications that demand high durability.

## 1. Introduction

Hydrogels, as polymeric networks capable of binding and retaining large amounts of water, have gained significant attention in the last couple of decades due to their high water content, excellent biocompatibility, and the considerable progress made in designing, synthesising, and utilising them for various biological and biomedical applications [1]. Their key characteristics, like cross-linking, shape adaptability, and elasticity, make them well-suited for innovative and essential uses in different branches of biomedicine [2]. Many of these hydrophilic polymer networks have a strong affinity for water, but their dissolution is prevented by their chemically and/or physically cross-linked structure [1,3,4,5,6].

Gelatin (G) is widely used in bio-hydrogel preparation due to its biocompatibility, non-toxicity, biodegradability, and water solubility, making it ideal for biomedical applications like drug delivery and tissue engineering. Its versatility in gelling, stabilising, thickening, emulsifying, and film formation makes it valuable for medical and pharmaceutical products [7,8,9,10]. The elasticity and flexibility of gelatin-based hydrogels can be improved by combining them with eco-friendly materials. However, gelatin’s brittleness and low strength can limit its use. Cross-linking with chemical reagents enhances mechanical properties by forming stronger covalent bonds [11,12,13]. Based on reversible hydrogen bonding and ionic interactions, the cross-linking process enhances hydrogel stability. Key factors like component concentration, reaction time, temperature, and drying must be managed to prevent protein denaturation and control hydrogel degradation [14,15].

Tannic acid (TA), a polyphenolic acid found in various plant-based foods, has attracted significant attention in the biomedical field due to its unique antiviral and antibacterial properties. Recognised as an approved food additive by the US Food and Drug Administration (FDA) [8,16,17], TA has been the subject of numerous studies highlighting its wide range of pharmacological activities, including anti-inflammatory, neuroprotective, antitumor, cardioprotective, and antipathogenic effects [18]. These multifaceted benefits make TA a promising compound for therapeutic applications. Extensive research has explored its application as an additive to biopolymer materials, taking advantage of its dual capabilities of antimicrobial and antiviral activity and its potential to enhance biological processes such as tissue regeneration and wound healing. Tannic acid has been successfully incorporated into various biopolymers, including collagen, chitosan, agarose, and starch. Due to polyhydroxy groups, TA can act as a cross-linker in two ways: physically or chemically, enabling the formation of versatile polymeric networks. In physical cross-linking, TA interacts with macromolecules at multiple binding sites via hydrogen bonding, ionic bonding, and π–π or cation–π interactions. Beyond these non-covalent interactions, TA is also capable of forming covalent bonds with functional molecules, resulting in chemically cross-linked polymeric networks [19]. Notably, recent studies have demonstrated tannic acid’s excellent binding affinity to biopolymers, along with its proven biological efficacy [20]. Various methodologies have been developed, including modifying gelatin hydrogels with TA using NaIO_4_ under basic conditions [21], followed by incorporating gelatin, glutaraldehyde, glycerol, and TA [22]. Another approach involves the incorporation of polyacrylic acid/tannic acid and the cross-linker 1,6-hexanediol bis(2-methyl-1-propionic acid azide), showcasing diverse strategies to enhance material properties through chemical modifications [23].

Pluronic F-127, an FDA-approved triblock copolymer, is widely accepted in pharmaceutical applications. Composed of hydrophilic polyoxyethylene (PEO) and lipophilic polyoxypropylene (PPO), it self-assembles into nanomicelles in water, aiding in hydrophobic drug delivery. Its thermosensitivity, injectability, biocompatibility, and biodegradability make it ideal for various biomedical uses. Pluronic F127 is a thermosensitive polymer. When heated to around 37 °C (body temperature), the PPO block becomes dehydrated and interacts with the PEO blocks to form spherical microgels. These microgels then link together to create a porous, three-dimensional hydrogel network, which can return to a liquid state when cooled to 4 °C. Once in gel form, it adheres well to tissues and provides sustained drug release over time. Polymeric micelles can transport several drugs, helping to extend their circulation time and enhance the permeability and retention effect. However, F127 and its derivatives are often not used on their own, as they typically require the addition of other materials to improve their mechanical strength and functional performance [24,25,26]. Cross-linkers are often added to improve hydrogels’ mechanical strength, stability, and overall functionality. Various methodologies have been developed to cross-link Pluronic F-127 with a variety of polymers from diverse sources, including different types of gelatin (type A, GA, and type B, GB) [27], alginate [28], gelatin–lecithin composites [29], gellan gum [30], among others.

Phytic acid (PA), or inositol hexaphosphate, is the most abundant inositol phosphate found in nature and plays a key role in various biological functions. Its unique structure, with several reactive phosphate groups, gives the PA a high negative charge density, which provides it with strong chelating ability and antioxidant properties. These hydroxyl-bearing phosphoric groups enable phytate to interact with a variety of substances, including cations, charged amino acids, and both charged and uncharged regions of polysaccharides. PA forms stable complexes with different biomolecules through electrostatic interactions and hydrogen bonding, making it a highly versatile compound in biological and material systems [31,32]. It is a non-toxic, plant-based molecule with antioxidant and antibacterial properties. It chelates Fe^3+^ ions and enhances hydrogel stability and biological performance while remaining safe, making it ideal for biomedical applications where safety and efficacy are paramount [31,33,34,35]. A variety of procedures have been developed to cross-link PA with various polymers, such as carboxymethyl cellulose [34], chitosan [36], polyaniline [37], poly((trimethylamino)ethyl methacrylate chloride) [35], poly(vinyl alcohol) [38], alginate [39], and polyacrylamide/chitosan [40]. The versatility of PA in forming stable, biocompatible networks with different polymers makes it a promising candidate for innovative therapeutic and diagnostic solutions.

Our recent study presented the synthesis of advanced hydrogels by physical cross-linking of gelatin, Pluronic F-127, and phytic acid [41]. The properties of hydrogels were dependent on the weight ratio of individual components. The most stable hydrogel was achieved by incorporating a higher amount of F-127 and PA with a weight ratio of G:F-127:PA = 3:2:1. These findings were also supported by the results of thermogravimetry and differential scanning calorimetry, which indicated a higher stability of this hydrogel compared to other hydrogel samples. FTIR spectra confirmed the formation of intermolecular bonding between the three components. Overall, the hydrogel with a higher amount of F-127 and PA was more stable and achieved a higher degree of swelling due to a highly porous network with fine and interconnected pores. In the present study, we continue our previous study [41] by introducing tannic acid in the synthesis to improve the hydrogel’s structural integrity and mechanical strength. Using naturally derived cross-linking agents enhances safety in biomedical applications while promoting innovation and environmental sustainability. This study highlights the crucial role of TA in the four-component hydrogel formulation. The incorporation of TA presents the novel hydrogel with enhanced overall stability, improved swelling capacity, and a higher melting point, exceeding physiological temperature (37 °C).

## 2. Materials and Methods

### 2.1. Chemicals and Materials

Gelatin from bovine skin, tannic acid, Pluronic^®^ F-127, and phytic acid sodium salt hydrate were obtained from commercial suppliers (Sigma Aldrich, St. Louis, MO, USA; Sigma-Aldrich Chemie GmbH, Steinheim, Germany; Merck, Darmstadt, Germany). All chemicals were used without any purification.

### 2.2. Hydrogel Formation

Selected ratios of gelatin, tannic acid, Pluronic F-127, and phytic acid were tested to determine the optimal conditions for hydrogel formation, as outlined in Table 1. The components were combined in 25 mL reactor tubes and then diluted with deionised water. The tubes were tightly sealed and heated to 65 °C (Mettler-Toledo Easymax 102 Advanced Synthesis Workstation, Columbus, OH, USA). The solution was continuously stirred for one hour using a magnetic stirrer to create a homogeneous solution. Then, 5 mL of the still-warm final mixture was transferred to a microplate, where the formed hydrogels were left at room temperature to cool down. The prepared hydrogels were subsequently either lyophilised or air-dried under ambient conditions (as xerogels) for characterisation.

### 2.3. Characterisation

#### 2.3.1. FTIR Spectroscopy of Reagents and Xerogel

The Fourier-transform infrared (FTIR) spectra of the reagent’s gelatin, copolymer F-127, phytic acid, and tannic acid (used as a reference), along with the xerogel, were recorded using a PerkinElmer Spectrum 100 instrument (Waltham, MA, USA) equipped with a universal attenuated total reflection (UATR) sampling accessory. The spectra were collected from 4000 to 500 cm^−1^, with a resolution of 4 cm^−1^, averaging four scans.

#### 2.3.2. Swelling Capacity of Xerogels

The xerogels (samples) were evaluated for their swelling capacity by immersing them in 0.1 M phosphate-buffered saline solution (PBS) at pH 7.4. The optimal one was also tested in phosphate buffer (PB) (Na_2_HPO_4_, KH_2_PO_4_) at a slightly acidic pH of 6.0 and a slightly more alkaline solution of 8.0 to find a correlation vs. solution pH. The samples were periodically weighed up to 74 h. After this, the swollen hydrogels were removed from the solution, and the excess liquid was gently removed by placing the sample on the filter paper before weighing. The swelling degree (Sw) given in % of the sample was then calculated using the following equation:

Sw = (W_t_ − W_0_)/W_0_ × 100 (1)

where W_0_ and W_t_ are the weights of the dry and wet samples, respectively.

#### 2.3.3. Scanning Electron Microscopy Investigation of Lyophilised Hydrogels

Scanning electron microscopy (SEM) imaging was conducted on lyophilised hydrogels. Liofilisation was performed under consistent conditions regarding freezing time, method, vacuum pressure, temperature, and other relevant parameters to identify potential morphological differences. The prepared lyophilised hydrogel was cut into small square sections using a sharp knife to obtain a well-defined sample cross-section a few millimetres in size. Before SEM imaging, the samples were sputter-coated with a carbon layer using a BAL-TEC SCD 005 sputter coater (BAL-TEC, Los Angeles, CA, USA). The surface, particularly along the cross-section, was examined with field emission scanning electron microscopy (FEI Helios NanoLab 600 Dual-beam, Hillsboro, OR, USA). Imaging was performed in secondary electron imaging (SEI) mode at 5 kV.

#### 2.3.4. Thermal Analysis of Xerogels and Lyophilised Hydrogels

The thermogravimetric (TG) measurements were carried out with a TGA/DSC1 instrument from Mettler Toledo (Greifensee, Switzerland). An amount of approximately 9 mg of the sample was placed in a 150 µL platinum crucible, isothermally treated for 10 min at 25 °C in an argon atmosphere at a flow rate of 100 mL/min, and then heated to 180 °C at a heating rate of 10 °C/min. An empty crucible served as a reference. The blank curve was subtracted. The evolved gases were channelled via a 75 cm long heated capillary into the coupled Pfeiffer Vacuum ThermoStar mass spectrometer (Asslar, Germany) for TG-MS measurement.

Differential Scanning Calorimetry (DSC), Mettler Toledo DSC1, Greifensee, Switzerland, was used to determine the melting properties of all samples. The instrument was calibrated using the melting enthalpy and melting temperature of MilliQ water and the indium reference standard distributed by Mettler Toledo. Approximately 4.5 mg of the sample was placed in a 40 μL aluminium pan (Mettler Toledo), weighed on an external Mettler Toledo MX5 balance, and hermetically sealed. An empty aluminium pan was used as a reference. The following measurement protocol was used: cooling from 25 °C to 10 °C at a rate of 2 °C/min, isothermal for 5 min, and heating from 10 °C to 55 °C at 2 °C/min under an argon flow of 100 mL/min. The enthalpy of melting was determined by integrating the peak area.

## 3. Results and Discussion

**Figure 1:**
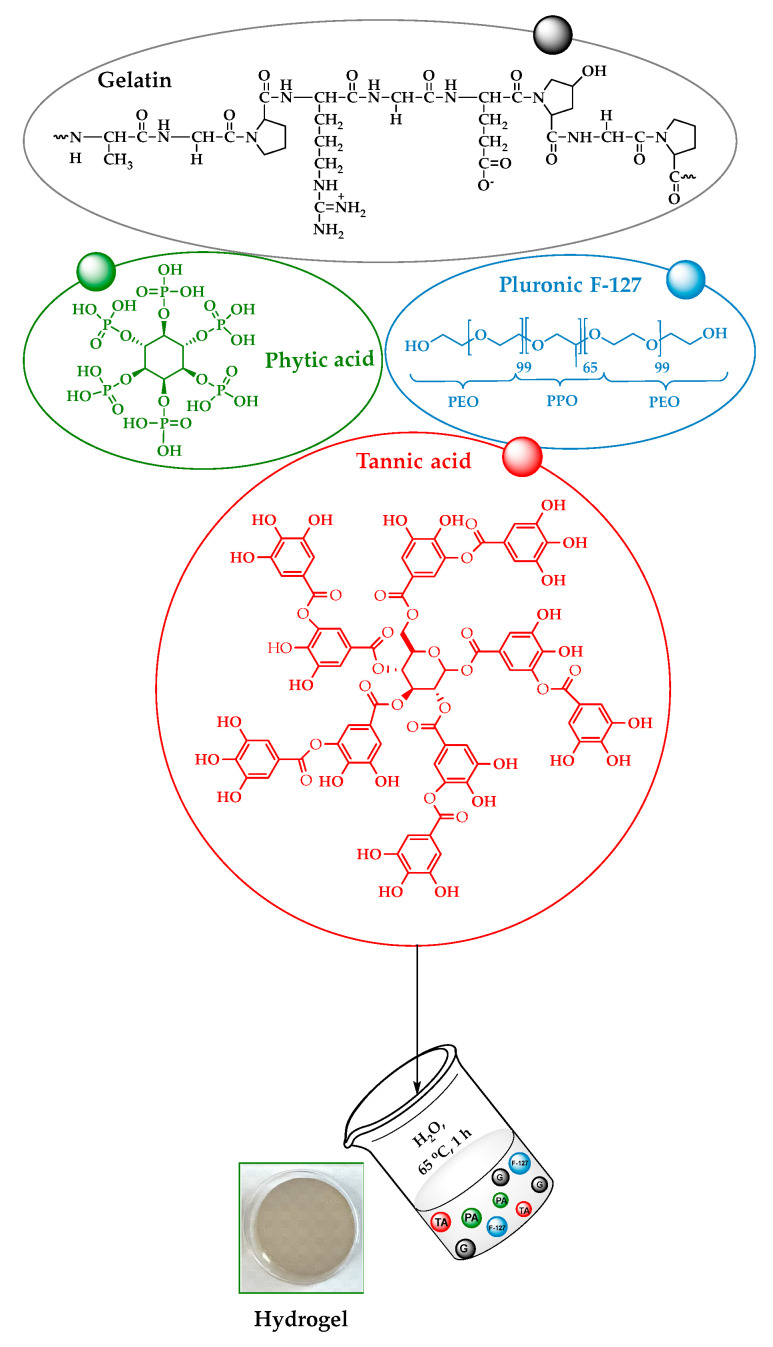
Flowchart for preparation of hydrogels consisting of gelatin (G), tannic acid (TA), triblock polymer Pluronic F-127 (F-127), and/or phytic acid (PA).

Hydrogel samples were prepared by varying the content of gelatin (G), tannic acid (TA), Pluronic F-127 (F-127), and phytic acid (PA) components in the mixture, as described in Table 1 and Figure 1.

**Figure 2:**
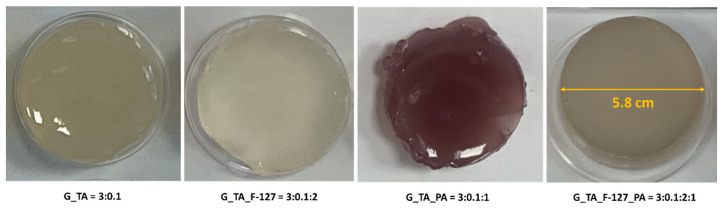
Optical images of the formed hydrogels after 24 h of cross-linking at ambient temperature. G_TA and G_TA_PA are robust and adhesive. G_TA_F-127 shows poor water absorption. G_TA_F-127_PA is smooth and flexible.

Figure 2 illustrates various formulations of freshly prepared hydrogels after 24 h of cross-linking at room temperature. The formulation only from G and TA (G_TA) results in a robust and adhesive hydrogel that lacks smoothness and has low flexibility. Adding F-127 (G_TA_F-127) does not allow the hydrogel to fix well in the microplate, leaving part of the water unabsorbed. When PA is added (G_TA_PA), the hydrogel remains robust and adhesive but still lacks smoothness and exhibits no flexibility. However, the combination of F-127 and PA added to G and TA (G_TA_F-127_PA) significantly enhances the hydrogel’s smoothness and flexibility compared to other formulations. Notably, precipitation occurred in the mixture when a higher amount of TA was used (i.e., ≥0.05 g). Based on the results presented in Section 3.2, Section 3.3 and Section 3.4, the G_TA_F-127_PA hydrogel formulation was identified as the best-performing among the other combinations (G_TA, G_TA_F-127, and G_TA_PA).

### 3.1. FTIR Spectroscopy Analysis

**Figure 3:**
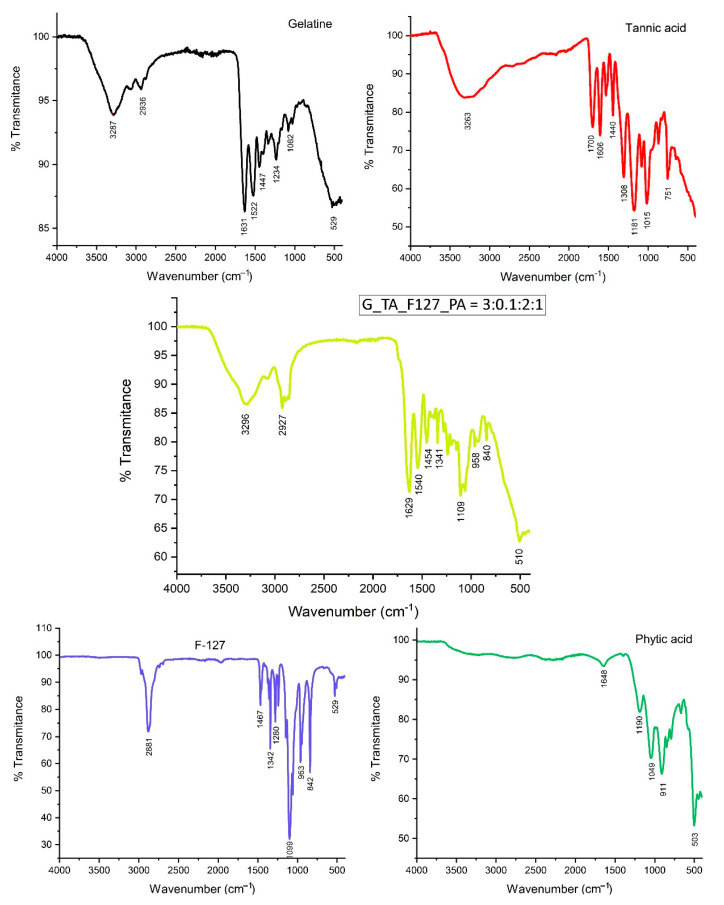
FTIR spectra of the hydrogel G_TA_F-127_PA along with the initial reagents (G, TA, F-127, and PA).

Figure 3 illustrates the FTIR spectra of reagents gelatin, TA, F-127, PA, and the hydrogel G_TA_F-127_PA. The IR spectrum of the latter displays characteristic bands from each of its constituent components, highlighting their contribution to the formation of the hydrogel network. A significant observation is the shift to a higher wavelength in the region around 3330 cm^−1^, linked to OH and NH_2_ stretching vibrations, which suggests the formation of intermolecular bonds between gelatin and/or F-127, TA, and PA. Furthermore, the hydrogel spectrum shows the presence of the C=O group band at 1625 cm^−1^, originating from gelatin. The band corresponding to P–O groups at 1188 cm^−1^ in PA shifts to a higher wavelength of 1239 cm^−1^ in the hydrogel, suggesting the formation of intermolecular bonds between gelatin and/or F-127 with PA.

### 3.2. Swelling Test of Xerogel Samples

**Figure 4:**
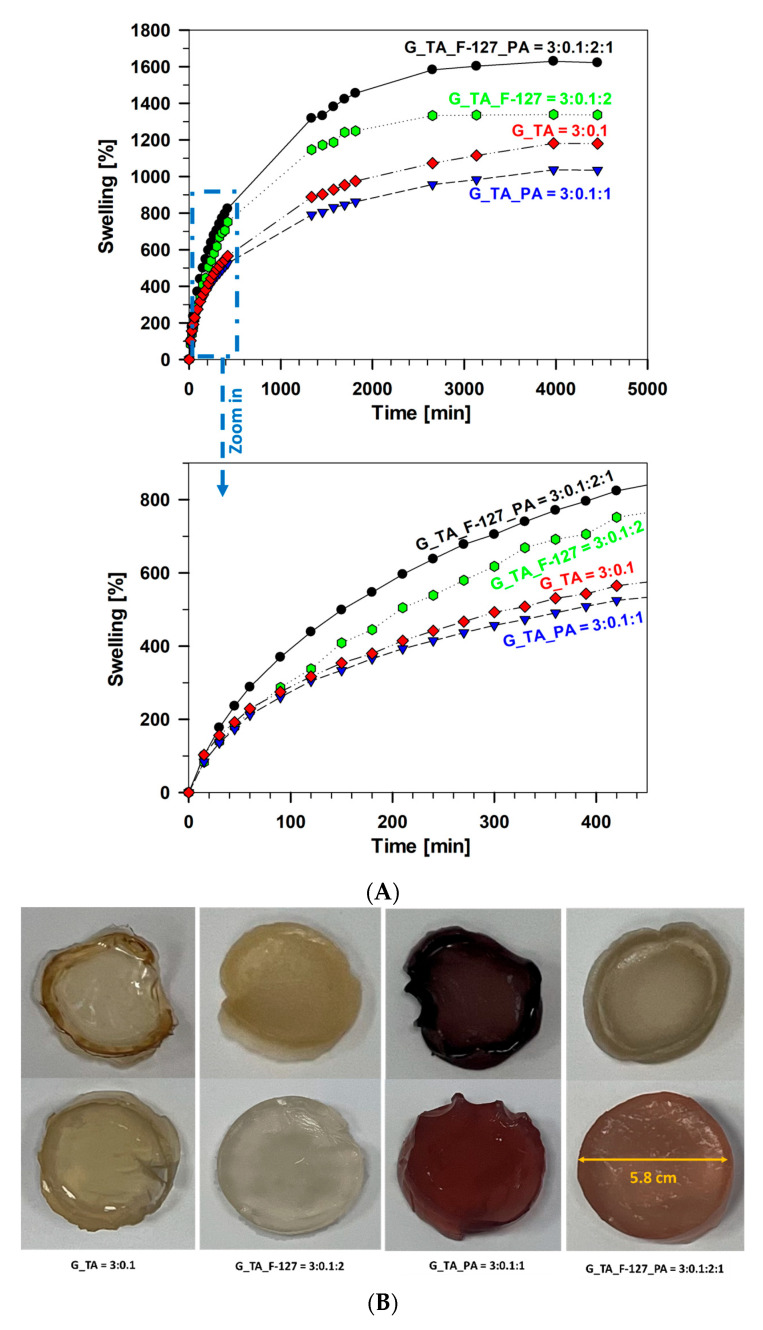
(**A**) The swelling percentages of the investigated hydrogel samples: G_TA_F-127_PA, G_TA_F-127, G_TA, and G_TA_PA in phosphate buffer solution (PBS, 0.1 M) at a pH of 7.4. (**B**) Images of xerogels after seven days of drying at room temperature (upper row) and a four-day swelling process in a 0.1 M PBS buffer at 30 °C after 4 days (Figure 4B, lower row). The ratios of components G, TA, F-127, and PA are given in the figure legend.

Figure 4A illustrates the swelling percentage of all tested hydrogel samples over time in a 0.1 M PBS solution at a pH of 7.4. The degree of swelling is influenced by the presence of cross-linkers, specifically the relative amounts of G, F-127, PA, and TA. Notably, lower swelling was observed in samples lacking F-127 (G_TA, 1180%, and G_TA_PA, 1035%). In contrast, the sample G_TA_F-127_PA demonstrated the highest swelling percentage in the final stages, reaching 1622%, and showed a greater degree of swelling than G_F-127_PA (1544%) and G_TA_F-127 (1337%). Additionally, the sample G_TA_F-127_PA exhibited a faster swelling rate (as highlighted in the zoomed section of Figure 4A). In all cases, swelling signs of saturation were evident, reaching a plateau after four days. A slight decrease in swelling accompanied this plateau. Including all four components (G, F-127, PA, and TA) resulted in a tightly interacting polymer network, as confirmed by SEM analysis, shown in the following section. The porous surface structure facilitated water movement through the polymer, enhancing the hydrogel’s swelling capacity. After being dried at room temperature for seven days until reaching a constant weight, the hydrogel samples were converted into xerogels (Figure 4B, upper row). Following a four-day swelling process in PBS buffer, the xerogels expanded and swelled (Figure 4B, lower row).

It appears that the sample G_TA_F-127 resulted in a lower degradation rate. Additionally, the samples G_TA_PA and G_TA showed weakened structures during the swelling process, making them highly prone to rupture when handled. In contrast, the hydrogel containing all four components (G_TA_F-127_PA) was the most stable, maintaining its cylindrical shape throughout. This stability suggests that the balanced incorporation of the components enhances the mechanical integrity of the hydrogel during swelling.

**Figure 5:**
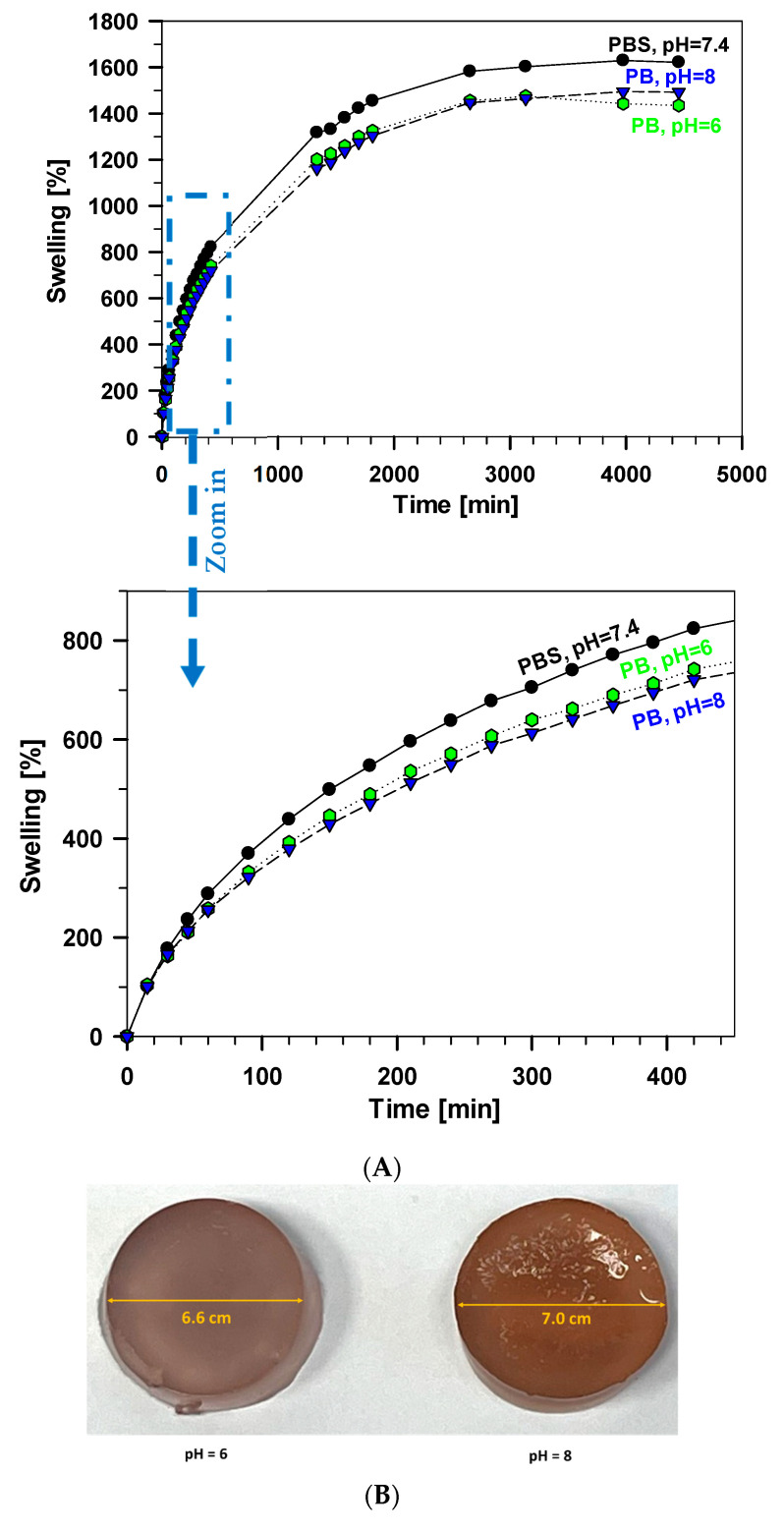
(**A**) The swelling percentages of the sample G_TA_F-127_PA in PBS and different pHs of phosphate buffer (PB). The lower graph enlarges the initial 400 min in the upper graph. (**B**) Images of swollen xerogel G_TA_F-127_PA after 4 days of swelling in phosphate buffer solutions at pHs of 6 and 8 at 30 °C.

The investigated samples displayed different absorption capacities in response to changes in the pH of the surrounding solution (Figure 5A). The hydrogel G_TA_F-127_PA exhibited the highest swelling degree at pH 7.4, which decreased at both acidic (pH 6, 1443%) and basic (pH 8, 1495%) conditions while still maintaining its cylindrical shape more effectively than at pH 7.4. This behaviour is attributed to the pH sensitivity of the hydrogel’s primary components: gelatin, F-127, phytic acid, and tannic acid. At pH 7.4, the hydrogel swells the most because its components interact well with water. Gelatin gains a positive charge at pH 6 (acidic), making the structure tighter and reducing swelling. At pH 8 (basic), some groups lose hydrogen, creating stronger bonds that hold the structure together, limiting swelling but keeping its shape. As a result, distinct swelling patterns emerged depending on the pH of the environment (Figure 5B). These findings suggest that the investigated hydrogel is pH-sensitive and thus can be adapted for various applications where pH conditions are crucial.

### 3.3. Structure Investigation Using Scanning Electron Microscopy

**Figure 6:**
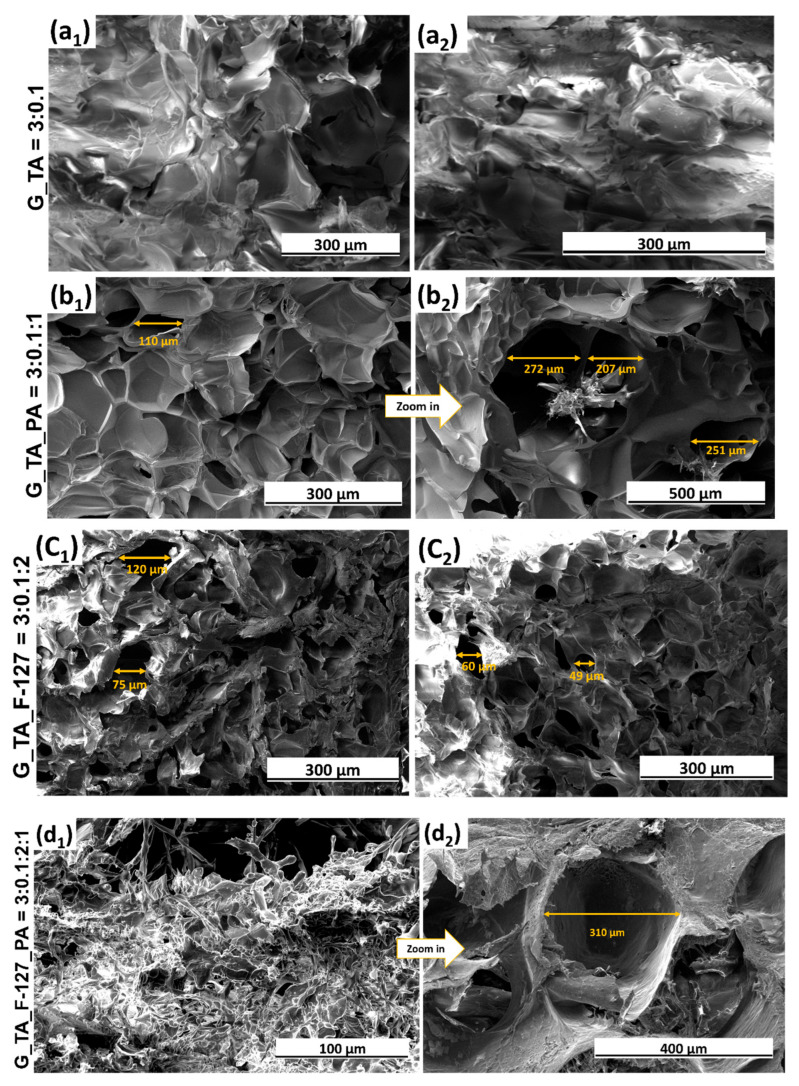
Secondary electron (SE) SEM images of lyophilised samples: (**a1**,**a2**) G_TA; (**b1**,**b2**) G_TA_PA; (**c1**,**c2**) G_TA_F-127; (**d1**,**d2**) G_TA_F-127_PA. The ratios of components G, TA, F-127, and PA are given in the figure legend.

The G_TA_F-127_PA lyophilised samples were investigated using SEM (Figure 6). The pore size and organisation varied across the samples. Pore size evaluations were taken from randomly selected pores, with images captured at different magnifications for analysis.

**Figure 7:**
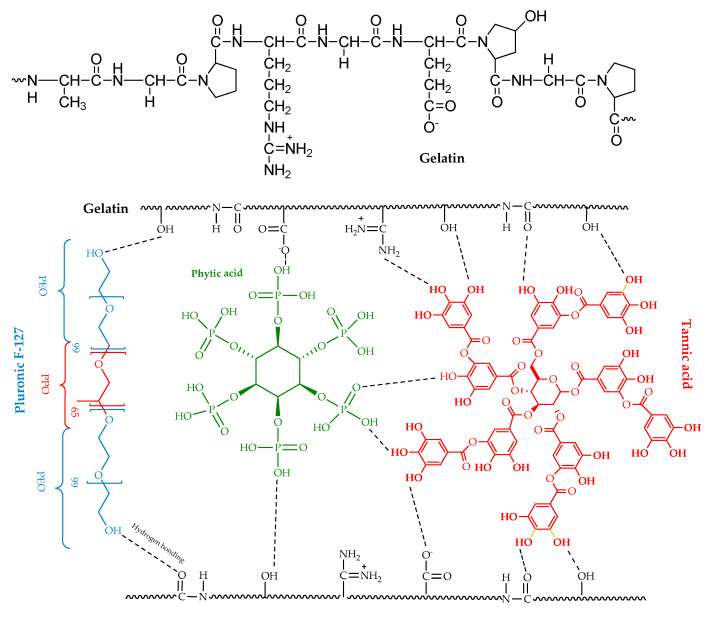
Schematic representation for hydrogel formation utilising gelatin, Pluronic F-127, phytic acid, and tannic acid.

Hydrogels without F-127 and PA exhibited a rough surface with only a few visible pores, as seen in Figure 6(a1,a2), indicating limited porosity and structural uniformity. When only F-127 was absent (Figure 6(b1,b2)), the hydrogels displayed disorganised pores of varying sizes, suggesting inconsistent pore formation. The pore size ranged from about 100 to 300 μm. Similarly, hydrogels without PA (Figure 6(c1,c2)) also showed disorganised pores but with thicker boundaries, highlighting a difference in the internal structure compared to the other samples. However, the hydrogels formed a highly porous, interconnected structure when all four components were incorporated, as shown in Figure 6(d1,d2). This combination of F-127, PA, and TA led to a more densely interconnected polymer network, resulting in hydrogels with more pores and greater overall porosity than those lacking one or more components. This enhanced structure suggests that all four components are essential for optimal pore formation and hydrogel network connectivity. Notably, the addition of TA, rich in hydroxyl (–OH) groups, results in intensified hydrogen bonding and other electrostatic interactions between the components, significantly enhancing the stability of the hydrogel network (Figure 7). A hydrogen bond is a weak electrostatic attraction between a hydrogen atom, covalently bonded to an electronegative atom (such as oxygen, nitrogen, or fluorine), and another electronegative atom with a lone pair of electrons. The hydroxyl groups in tannic acid interact with the carbonyl (C=O) and amine (NH) groups in gelatin, forming hydrogen bonds. Additionally, TA forms hydrogen bonds with the phosphate (–PO_4_) and hydroxyl (–OH) groups of PA. Furthermore, the hydroxyl groups in G can establish hydrogen bonds with the oxygen atoms in the polyethylene oxide (PEO) chains of F-127.

**Figure 8:**
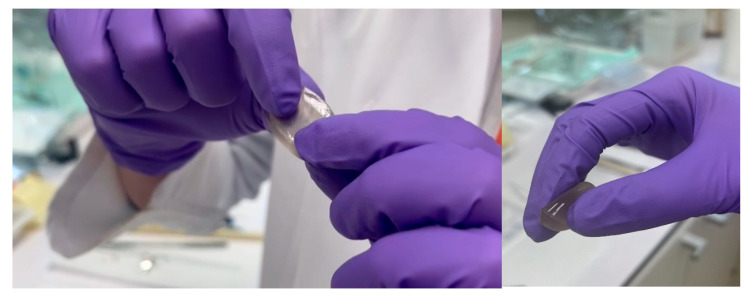
Elasticity evaluation of the hydrogel G_TA_F-127_PA = 3:0.1:2:1.

This incorporation of TA enhances the cross-linking efficiency, strengthening the overall structure compared to hydrogels without TA [39] (Figure 8). A demonstration of the hydrogel’s elasticity assessment is available in Video S1, provided in the Supplementary Materials.

### 3.4. Thermal Analysis

**Figure 9:**
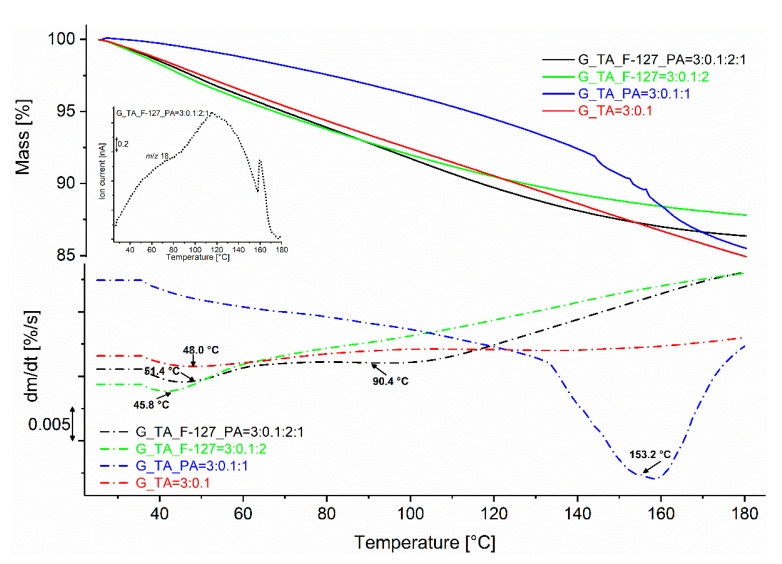
Dynamic TG (line) and DTG (dash-dot) curves in argon of the xerogel samples between 25 °C and 180 °C. The embedded graph shows the MS curve of the evolved water during the TG measurement of the sample G_TA_F-127_PA.

Dynamic thermogravimetric (TG) measurements of the xerogels in argon showed (Figure 9 upper panel) that for all samples except G_TA_PA, there is a fairly uniform mass loss over the entire temperature range up to 180 °C, which is due to the dehydration of the xerogels, as can be seen from the embedded TG-MS evolved gas analysis graph of sample G_TA_F-127_PA, which shows a characteristic signal for water (*m/z* = 18) and for the hydroxide fragment 17 (not shown in the graph). The temperature range in which the maximum rate of mass loss is observed was determined by the first derivative of the TG curve (DTG curves in Figure 9, lower panel). The greatest mass loss was observed in the samples without F-127 cross-linker, G_TA_PA and G_TA (Table 2), at 14.8% and 15.3%, respectively, where minor or irregular porosity was observed (Figure 6). The course of the mass loss of the otherwise rigid sample G_TA_PA, which also exhibited the worst swelling properties, is slower than all other samples up to 130 °C, i.e., in the range in which the maximum mass loss due to water is to be expected. This is followed by an abrupt mass loss up to 180 °C, with an inflection point at 153.2 °C. This effect is not observed in the lyophilised corresponding sample (Figure S1), which indicates that the water requires more thermal energy to be released from the pores of the inflexible structure of the sample.

All other prepared xerogels show a maximum mass loss rate of up to 70 °C (Figure 9, Table 2). The sample G_TA_F-127_PA has an additional increased range of mass loss between 80 °C and 125 °C with an inflection point at 90.4 °C and a total mass loss of 13.7% to 180 °C, which is 1.5% more than the other sample with the cross-linker F-127, G_TA_F-127.

For all corresponding lyophilised analogues, the mass loss is only observed in a uniform but broader temperature range of 40–120 °C with an inflection point around 60 °C and is reduced by 40% for samples with the cross-linker F_127, or 50% if no PA is present, and by almost 30% for both samples without F-127 (see Figure S1 and Table S1).

**Figure 10:**
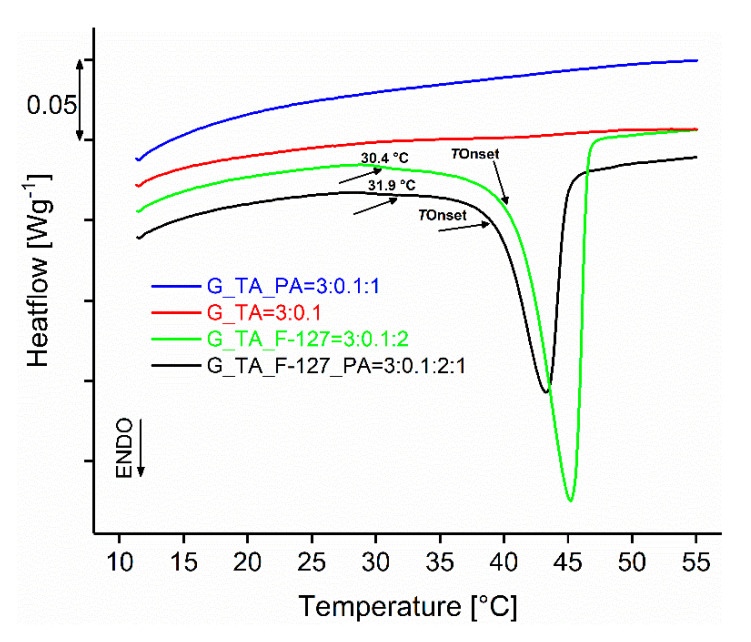
DSC curves in argon of the xerogel samples. *T*_Begin_ of melting is indicated.

Neither the xerogel nor lyophilised samples without F-127 cross-linker (G_TA and G_TA_PA) show any thermal effects on the DSC curves in the temperature range between 10 °C and 55 °C (see the flat baselines in Figure 10 and Figure S2), while the xerogel samples G_TA_F-127 and G_TA_F-127_PA show single narrow endothermic peaks with *T*_Onset_ temperatures at 40.9 °C and 39.4 °C, respectively (Table 3). At favourable temperatures of 30.4 °C and 31.9 °C, which are 10.5 °C and 8.5 °C below the *T*_Onset_ temperatures, respectively, the temperature of the beginning of the melting interval can be determined visually, as the DSC curve deviates from the baseline (*T*_Begin_), which also means that the samples will melt at human body temperature. The determination of the enthalpy of melting shows that G_TA_F-127_PA, the sample with the most uniform pore distribution (Figure 6) and the best result in the swelling test (Figure 4), has the lowest enthalpy value of 15.6 J/g, which is 38.3% lower than the sample without PA. In the lyophilised samples, the enthalpies of the two samples increase and are almost equal at 23.4 J/g and 27.4 J/g, respectively (Table S2, Figure S2). These findings indicate higher moisture content in the structure of sample G_TA_F-127_PA, as already determined by the TG-MS measurement (Figure 9), as water reduces the amount of heat required for the conversion of solid to melt. The latter is also shown by the increase in the *T*_Onset_ melting point temperatures for both lyophilised samples by about 6 °C compared to the original xerogels.

## 4. Conclusions

This study presented the synthesis of advanced (bio)hydrogels through the physical cross-linking of gelatin, tannic acid, Pluronic F-127, and phytic acid. Combinations of gelatin with (i) tannic acid (GA_TA), (ii) TA and F-127 (G_TA_F-127), (iii) phytic acid (G_PA), and (iv) all four components (G_TA_F-127_PA) were investigated. FTIR spectra confirmed the intermolecular interactions between the components, as evidenced by shifts in the absorption bands related to hydroxyl groups. All hydrogels demonstrated favourable swelling behaviour. The hydrogel containing all components was more stable and achieved higher degrees of swelling. The G_TA_F-127_PA hydrogel with the component weight ratios of 3:0.1:2:1 showed the highest swelling in phosphate saline buffer at a pH of 7.4 (1630%) and reduced swelling in acidic conditions of pH = 6 (1443%) and basic conditions of pH = 8 (1495%). The samples maintained their shape at pH 6 and 8, indicating that the hydrogel can be adapted for various applications where pH conditions are critical.

SEM revealed a highly porous structure with fine, interconnected pores, especially in the G_TA_F-127_PA sample. This porous structure enhances the hydrogel’s ability to absorb large amounts of water.

Thermal analysis confirmed that this specific hydrogel exhibits enhanced stability compared to other samples. This is proven by the narrowest melting temperature interval in the physiological range, the lowest melting enthalpy, and the highest water content determined by DSC and TG-MS measurements.

Overall, this system represents a significant step toward sustainable and safe biomaterial development. Using tannic acid and phytic acid as natural cross-linkers supports green chemistry principles, avoiding chemical agents and reducing potential cytotoxicity. These findings demonstrate the hydrogel’s promising potential for various biomedical and industrial applications. Future research will focus on applying these innovative hydrogels for wound-healing applications.

## Figures and Tables

**Table 1:** The weight of compounds used for hydrogel preparation and the corresponding sample codes. Reaction condition: T = 65 °C; t = 1 h, followed by cross-linking at ambient temperature for 24 h. The best-performing hydrogel combination is bolded.

Gelatin (g)	Tannic Acid (g)	Pluronic F-127 (g)	Phytic Acid (g)	Sample Abbreviations and Component Ratios	Short Abbreviation
0.3	0.01	/	/	G_TA = 3:0.1	G_TA
0.3	0.01	0.2	/	G_TA_F-127 = 3:0.1:2	G_TA_F-127
0.3	0.01	/	0.1	G_TA_PA = 3:0.1:1	G_TA_PA
**0.3**	**0.01**	**0.2**	**0.1**	**G_TA_F-127_PA = 3:0.1:2:1**	**G_TA_F-127_PA**

**Table 2:** Mass loss and Inflection point temperatures (*T*_Inflect. Pt._) of the xerogel samples between 25 °C and 180 °C in argon.

Sample Designation and Component Ratios	Mass Loss %	*T*_Inflect. Pt._ (°C)
G_TA = 3:0.1	15.3	48.0
G_TA_F-127 = 3:0.1:2	12.2	45.8
G_TA_PA = 3:0.1:1	14.8	153.2
G_TA_F-127_PA = 3:0.1:2:1	13.7	51.4 and 90.4

**Table 3:** Melting *T*_Begin_ and *T*_Onset_ temperatures and the corresponding enthalpies of the xerogel samples.

Sample Designation and Component Ratios	*T*_Begin_ (Melting) (°C)	*T*_Onset_ (Melting) (°C)	Δ*H* (Jg^−1^)
G_TA = 3:0.1	/	/	/
G_TA_F-127 = 3:0.1:2	30.4	40.9	25.3
G_TA_PA = 3:0.1:1	/	/	/
G_TA_F-127_PA = 3:0.1:2:1	31.9	39.4	15.6

## Data Availability

All data and materials are available on request from the corresponding authors. The data are not publicly available due to ongoing research using part of the data.

## Supplementary Materials

The following supporting information can be downloaded at: https://www.mdpi.com/article/10.3390/polym17101372/s1, Figure S1: Dynamic TG (dot) and DTG (dash-dot) curves in argon of the lyophilised samples between 25 °C and 180 °C; Figure S2: DSC curves in argon of the lyophilised samples. *T*_Begin_ of meltings indicated; Table S1: Mass loss and Inflection point temperatures (*T*_Inflect. Pt._) of the lyophilised samples between 25 °C and 180 °C in argon; Table S2: Melting *T*_Begin_ and *T*_Onset_ temperatures and the corresponding enthalpies of the lyophilised samples; Video S1: Hydrogels.
