# Supplementary material for: Enhancing Cross-Linking Efficiency in Gelatin-Based Hydrogels via Incorporation of Tannic Acid, Pluronic F-127, and Phytic Acid
Source: Polymers (Basel). 2025 May 16;17(10):1372. doi: 10.3390/polym17101372 (PMC12115240; doi:10.3390/polym17101372)
Supplement: Supplementary file 1 [file polymers-17-01372-s001.zip › polymers-3642291-supplementary.pdf]

Enhancing Cross-linking Efficiency in Gelatin-based Hydrogels *via* Incorporation of Tannic Acid, Pluronic F-127, and Phytic Acid

Njomza Ajvazi [1,]* Ingrid Milošev [1,2], Nataša Čelan Korošin [3], Peter Rodič [1] and Bojan Božić [4]

[1] Jožef Stefan Institute, Jamova 39, 1000 Ljubljana, Slovenia; njomza.ajvazi@ijs.si (N.A.), ingrid.milosev@ijs.si (I.M.), peter.rodic@ijs.si (P.R.)
[2] Valdoltra Orthopaedic Hospital, Jadranska c. 31, 6280 Ankaran, Slovenia; ingrid.milosev@ob-valdoltra.si
[3] Faculty of Chemistry and Chemical Technology, University of Ljubljana, Večna pot 113, 1000 Ljubljana, Slovenia; natasa.celan@fkkt.uni-lj.si
[4] University of Belgrade-Faculty of Biology, Institute of Physiology and Biochemistry "Ivan Djaja", Serbia; bbozic@bio.bg.ac.rs
* Correspondence: njomza.ajvazi@ijs.si

Supporting Information

Table S1. Mass loss and Inflection point temperatures ($T_{\text{Inflect. Pt.}}$) of the lyophilised samples between 25 °C and 180 °C in argon.

Sample designation and component ratios	Mass loss %	$T_{\text{Inflect. Pt.}}$ (°C)
G_TA = 3:0.1_L	10.9	59.8
G_TA_F-127= 3:0.1:2_L	6.0	60.4
G_TA_PA = 3:0.1:1_L	10.7	65.0
G_TA_F-127_PA = 3:0.1:2:1_L	8.1	58.7

Table S2. Melting T_{Begin} and T_{Onset} temperatures and the corresponding enthalpies of the lyophilised samples.

Sample designation and component ratios	T_{Begin} (Melting) (°C)	T_{Onset} (Melting) (°C)	ΔH (Jg^{-1})
G_TA = 3:0.1_L	/	/	/
G_TA_F-127= 3:0.1:2_L	36.9	46.3	27.4
G_TA_PA = 3:0.1:1_L	/	/	/
G_TA_F-127_PA = 3:0.1:2:1_L	34.5	46.0	25.4

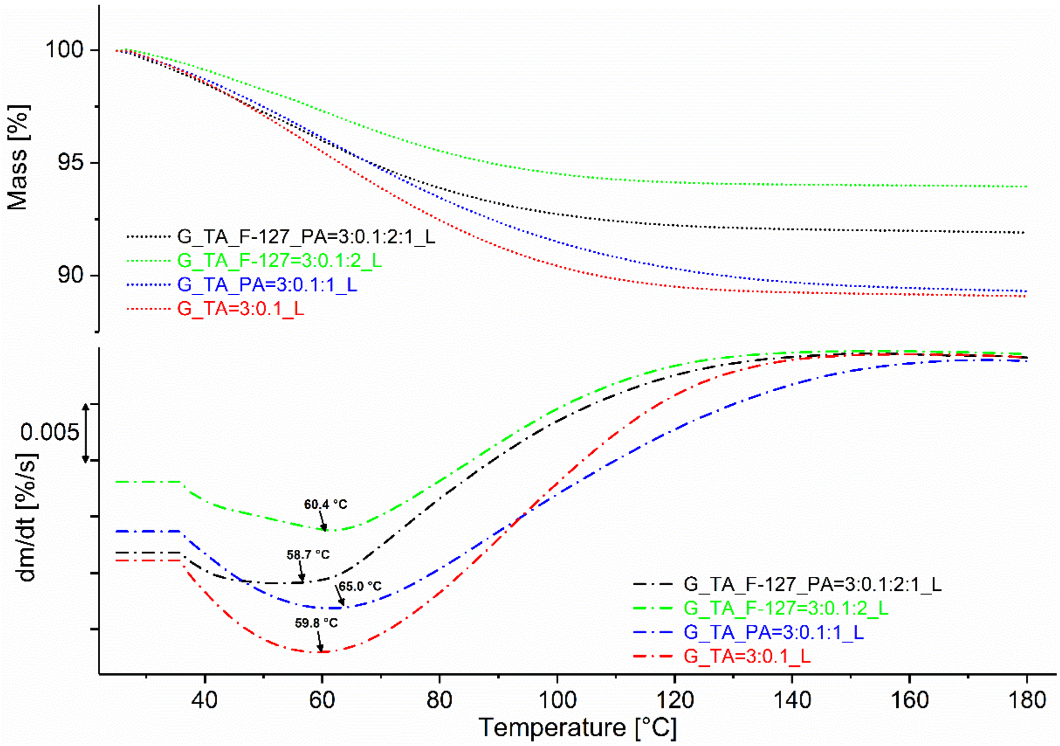

Figure S1. Dynamic TG (dot) and DTG (dash-dot) curves in argon of the lyophilised samples between 25 °C and 180 °C.

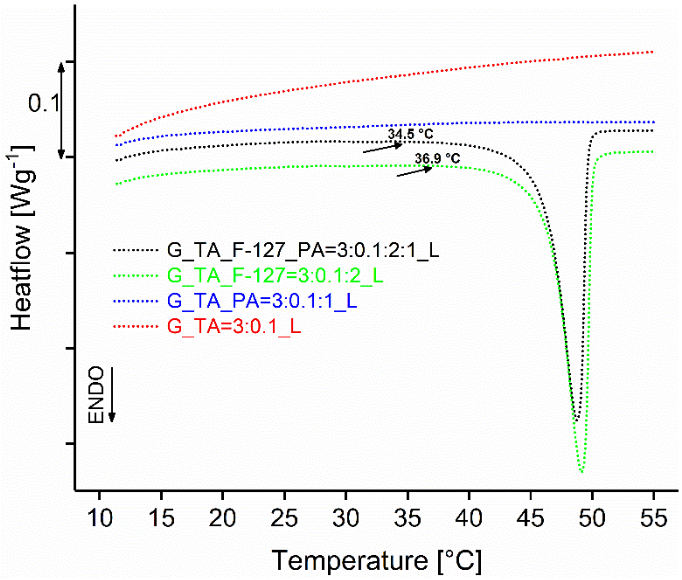

Figure S2. DSC curves in argon of the lyophilised samples. T_{Begin} of meltings indicated.
